# A case of cutaneous polyarteritis nodosa treated with baricitinib

**DOI:** 10.1016/j.jdcr.2025.11.013

**Published:** 2025-11-21

**Authors:** Hira Asim, Stephen Mason, Hina Tareen, Sanober A. Amin

**Affiliations:** a Long School of Medicine, The University of Texas Health Science Center at San Antonio, San Antonio, Texas; b Pathology Service, Independent Researcher, Flower Mound, Texas; c Rheumatology Service, Rheumatology & Autoimmune Specialists, Flower Mound, Texas; d Dermatology Service, Dermatology Solutions, Grapevine, Texas

**Keywords:** baricitinib, cutaneous polyarteritis nodosa, Janus kinase inhibitor, subcutaneous nodules

## Introduction

Cutaneous polyarteritis nodosa (cPAN) is a subtype of polyarteritis nodosa, a rare form of medium-sized vessel vasculitis, characterized by its disabling and prolonged disease course of subcutaneous nodules, livedo reticularis, and ulcerations that are mainly located on the lower extremities. Treatment of cPAN depends on disease severity as well as relapse frequency, but initial treatment for mild disease limited to skin generally consists of nonsteroidal anti-inflammatory drugs, colchicine, and topical glucocorticoids. For more severe cases, oral glucocorticoids, azathioprine, methotrexate, mycophenolate mofetil, and intravenous immunoglobulins are used.^1^ Baricitinib, an oral, selective Janus kinase (JAK) 1/JAK2 inhibitor is known for its efficacy in treating inflammatory skin diseases, but has not been widely used in the context of polyarteritis nodosa. In this case report, we describe a patient with refractory cPAN who experienced successful symptom resolution with baricitinib.

## Case report

A 58-year-old woman with a medical history of osteoarthritis and both cutaneous and systemic sarcoidosis involving the joints presented with a 1-year history of worsening muscle pain, fatigue, and one episode of high fever. She also had 3-month history of tender, dark nodules on her extremities that worsened with physical activity, worse by end of each day. Acetaminophen and ibuprofen were minimally helpful.

**Fig 1:**
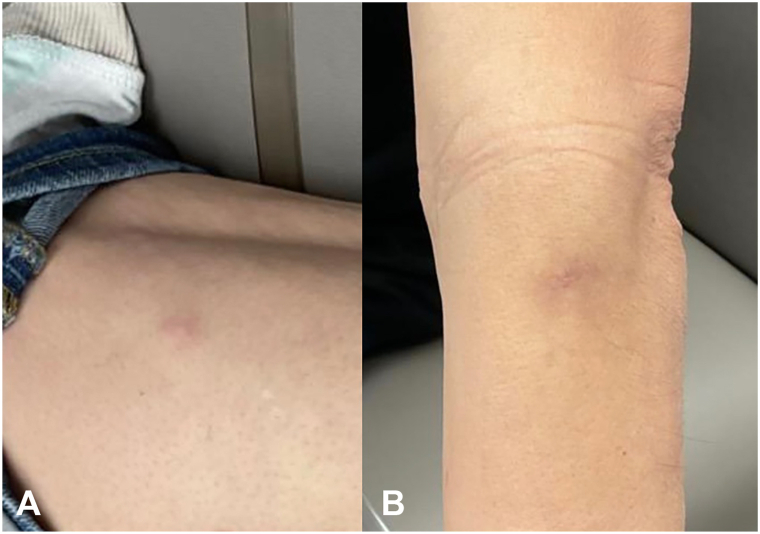
Clinical images of subcutaneous nodules. **A,** Lateral aspect of the left thigh nodule. **B,** Left elbow nodule.

Physical examination revealed tender, violaceous to brown papules and plaques involving the head, neck, trunk, and extremities, varying in size from a few millimeters to about a centimeter (Fig 1). Differential diagnosis included cutaneous sarcoidosis because of the patient’s medical history of sarcoidosis characterized by subcutaneous nodules similar to her current clinical presentation.

**Fig 2:**
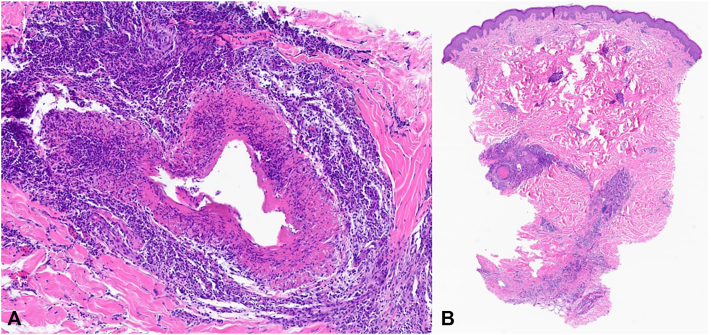
**A,** Biopsy with hematoxylin-eosin (H&E) revealing medium-sized arteriole with surrounding mixed inflammation and neutrophils within vascular smooth muscle. **(B)** Biopsy with H&E revealing deep medium-sized arteriole with surrounding dense lymphohistiocytic infiltrate and fibrin thrombus. (**A** and **B,** Hematoxylin-eosin stain; original magnifications: **A,** ×200; **B,** ×10.)

Laboratory test results included an autoimmune workup with negative antinuclear antibody, Smith/Ribonucleoprotein, SS-A/SS-B, double-stranded DNA, Ribosomal P, Scl70, rheumatoid factor/citric citrullinated peptide, antineutrophil cytoplasmic antibody, anticardiolipin, β-2 glycoprotein 1, and complement levels within normal range. Punch biopsy of the left elbow nodule revealed a medium-sized dilated arteriole with fibrinoid necrosis in the reticular dermis, neutrophils present both within the vascular wall and outside the wall, and a focal organizing clot (Fig 2). Features expected of sarcoidosis, such as noncaseating granulomas, were absent. Secondary to the inflammatory pattern, a periodic acid–Schiff stain was performed, which was negative for fungal organisms. Fite stain was negative for acid-fast organisms. Direct immunofluorescence was not performed.

The histologic findings were consistent with cPAN rather than sarcoidosis. The lack of positive autoimmune markers served to make a diagnosis of other types of vasculitis unlikely. In addition to the normal systemic workup and unremarkable imaging studies including abdominal and renal ultrasounds, the patient’s lack of systemic symptoms such as hypertension and abdominal pain favored a diagnosis of cPAN over systemic polyarteritis nodosa.

**Fig 3:**
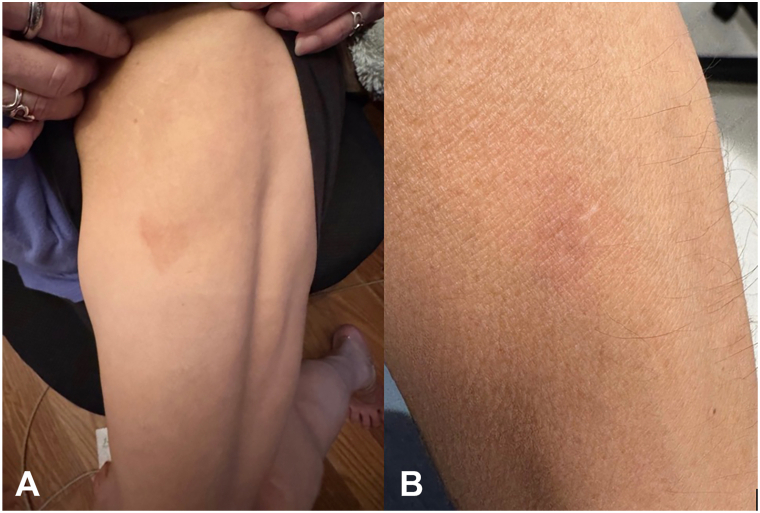
Clinical images of subcutaneous nodules after initiating treatment with baricitinib. **A,** Lateral aspect of the left thigh nodule significantly decreased in size. **B,** Left elbow nodule significantly decreased in size.

The patient was subsequently treated with intralesional triamcinolone injections, systemic steroids, mycophenolate mofetil, and duloxetine for neuropathic pain, with minimal control of her arthritis and periods of remission and relapse of cPAN. Although this combination had been the most successful medication regimen thus far, it did not result in complete pain relief, thus it was suggested that baricitinib, a JAK inhibitor known for its anti-inflammatory and immunomodulator properties, be trialed. Prednisone and duloxetine were continued per her rheumatologist but mycophenolate mofetil was discontinued. Within 1 to 2 days of taking baricitinib 2 mg, daily, patient reported complete pain relief in her lower extremities, but not cutaneous lesions. Prednisone was discontinued at this point. She was prescribed baricitinib 4 mg for the second month, and continued on duloxetine, which resolved her cPAN nodules. She has been on baricitinib as monotherapy for 8 months now without any recurrence of cPAN nodules or arthritis (Fig 3). Her surveillance laboratory tests, including lipid panel have been within normal limits.

## Discussion

We present the case of a 58-year-old woman diagnosed with cPAN based on clinical presentation and histopathologic evidence. Our patient experienced unremitting cPAN symptoms of painful subcutaneous nodules under treatment with various combinations of prednisone, mycophenolate, and duloxetine for over a year. However, after initiation of treatment with baricitinib, a JAK inhibitor known for its therapy in autoimmune inflammatory disorders, her arthritic symptoms resolved within days.

Considering the limited array of options for treating cPAN, as well as its chronic relapsing disease course, it is worthwhile to further explore JAK inhibitors’ role in treating this disease, given their ability to control multiple inflammatory and immune diseases.^2^ Although other JAK inhibitors, such as tofacitinib (5 mg, twice daily), have been reported to improve cPAN symptoms, there is minimal existing literature on the prescription of baricitinib for the treatment of cPAN.^3^^,^^4^

The remarkable clinical response observed in our patient may be attributed to the unique mechanism of JAK inhibition. JAK inhibitors block intracellular signaling downstream of several proinflammatory cytokines that drive vascular inflammation and endothelial injury in cPAN such as interleukins, interferons, and tumor necrosis factor-related mediators. In contrast, conventional immunosuppressants such as corticosteroids and antimetabolites act more broadly or upstream, which may not fully suppress cytokine-mediated activation within the vessel wall. By directly targeting the JAK–STAT pathway, baricitinib may have more effectively interrupted this inflammatory cascade, leading to rapid symptom resolution and sustained remission despite previous therapeutic resistance.

This case report exemplifies the relatively rapid and effective ability of baricitinib to reduce the symptoms of a patient with cPAN, specifically pain eradication and lesion shrinkage, and no further recurrence or relapse under a low dosage (2-4 mg, daily), while allowing for prednisone taper. In conclusion, baricitinib could be used as a first-line treatment option for disease-free remission in refractory cPAN once further studies have confirmed its efficacy.

## Conflicts of interest

None disclosed.
